# Ovarian Cancer Stem Cells with High ROR1 Expression Serve as a New Prophylactic Vaccine for Ovarian Cancer

**DOI:** 10.1155/2019/9394615

**Published:** 2019-03-17

**Authors:** Di Wu, Xiaoyu Yu, Jing Wang, Xu Hui, Yunxia Zhang, Yunlang Cai, Mulan Ren, Mei Guo, Fengshu Zhao, Jun Dou

**Affiliations:** ^1^Department of Pathogenic Biology and Immunology of Medical School, Southeast University, Nanjing 210009, China; ^2^Department of Gynecology & Obstetrics, Zhongda Hospital, Medical School, Southeast University, Nanjing 210009, China

## Abstract

Tumor vaccines offer a number of advantages for cancer treatment. In the study, the vaccination with cancer stem cells (CSCs) with high expression of the type I receptor tyrosine kinase-like orphan receptor (ROR1) was evaluated in a murine model for the vaccine's immunogenicity and protective efficacy against epithelial ovarian carcinoma (EOC). CD117^+^CD44^+^ CSCs were isolated from human EOC HO8910 cell line using a magnetic-activated cell sorting system; murine ID8 EOC suspension sphere cells, which are collectively known as cancer stem-like cells, were acquired from serum-free suspension sphere-forming culture. Mice were subcutaneously immunized with the repeat cycles of freezing and thawing whole HO8910 CD117^+^CD44^+^ CSCs and ID8 cancer stem-like cells, respectively, followed by a challenge with HO8910 or ID8 cells at one week after final vaccination. The results showed that the CSC vaccination significantly induced immunity against EOC growth and markedly prolonged the survival of EOC-bearing mice in the prophylactic setting compared with non-CSC vaccination. Flow cytometry showed significantly increased immunocyte cytotoxicities and remarkably reduced CSC counts in the CSC-vaccinated mice. Moreover, the protective efficacy against EOC was decreased when the ROR1 expression was downregulated by shRNA in CSC vaccines. The findings from the study suggest that CSC vaccines with high ROR1 expression were highly effective in triggering immunity against EOC in vaccinated mice and may serve as an effective vaccine for EOC immunoprophylaxis.

## 1. Introduction

Epithelial ovarian carcinoma (EOC) is the most prevalent form of ovarian cancer, causing more deaths than any other gynecologic malignancy [1, 2]. At present, the mainstay of EOC treatment consists of cytoreductive surgery and platinum-based chemotherapy. Though EOC is a highly chemosensitive disease, the disease is often diagnosed only at an advanced stage [3–5] and is therefore hard to cure. The majority of women with stage III/IV ovarian cancer who achieve clinical complete response with a frontline standard of care will relapse within 2 years [6]. This may be due to a subset of cancer stem cells (CSCs) that are relatively resistant to conventional chemotherapy and responsible for EOC metastasis and recurrence [7–9]. There is an urgent need for new treatment options that will be effective against such CSCs to improve EOC therapeutic efficiency and to extend ovarian cancer patients' survival.

Growing evidence has shown that the patients with gynecologic cancers, such as ovarian cancer, are in fact able to elicit endogenous antitumor immune responses and that these cancer patients may benefit from immunotherapy. Present approaches of active and passive immunotherapy for cancers include antibody-based therapies, immune checkpoint blockade, adoptive T-cell therapy, chimeric antigen receptor-modified T cells, and cancer vaccines [10, 11]. However, the results of immunotherapeutic vaccine approaches are still far below expectations due to the rarity of targetable tumor-specific antigens [11, 12]. Improved understanding of EOC biological features, immunological escape mechanisms, and signaling pathways has emerged in the past few years [12, 13]. Most studies of immunotherapy have suggested that the key to effective immunotherapeutic treatment involves novel agents as targeting therapies for CSC subset; such a treatment will benefit EOC patients [14, 15].

In a recent study, we have demonstrated that the human SKOV3 CD117^+^CD44^+^ CSC vaccination elicited strongly immune responses against ovarian cancer and significantly led to suppressing tumor xenografted growth in nude mice [16]. In the present study, we extended the previous investigation and developed the EOC CSC vaccines from human HO8910 CD117^+^CD44^+^ CSC line and murine ID8 EOC suspension sphere cells that were thought to be cancer stem-like cells [17, 18] in order to avoid the vaccine immunogenic deviation due to the different origin cells. Here, we showed that the EOC CSC vaccination induced a robust immune response against EOC cell challenge in a murine model. Furthermore, we found that the type I receptor tyrosine kinase-like orphan receptor (ROR1), a promising target for immunotherapy, was highly expressed in HO8910 CSCs and ID8 cancer stem-like cells and that knockdown of ROR1 via small interfering RNA (siRNA) in CSCs decreased the prophylactic efficacy of CSC vaccination. These results support that the high expression of ROR1 in CSCs closely correlates with the EOC CSC vaccine efficacy and CSC vaccine may serve as an immunotherapeutic candidate for ovarian carcinoma immunoprophylaxis.

## 2. Materials and Methods

### 2.1. Cell Lines

HO8910 cell line is from an ovarian cancer patient of origin, a well-established ovarian cancer model system. YAC-1 cell line is from Moloney leukemia-induced T-cell lymphoma; both cell lines were purchased from the Cell Bank of the Chinese Academy of Sciences (Shanghai, China). ID8, a clone of the MOSEC ovarian carcinoma of C57BL/6 origin was a gift from Dr. George Coukos (University of Pennsylvania, Philadelphia, USA). These cells are cultured at 37°C in a 5% CO_2_ atmosphere in RPMI 1640 supplemented with 10% fetal bovine serum (FBS), 25 mM HEPES, 2 mM glutamine, 100 U/mL penicillin, and 100 *μ*g/mL streptomycin sulfate.

### 2.2. Mice

The autosomal recessive Balb/c nude gene in homozygous (sp/sp) mice 6-7 weeks of age and C57BL/6 mice 6-7 weeks of age were purchased from the Animal Center of Yangzhou University of China (license number SCXK, Jiangsu province of China, 2007-0001). All the mice were maintained in a pathogen-free facility that has a 12-hour light/dark cycle and relative humidity ranging from 40% to 50% at 22°C.

### 2.3. Magnetic-Activated Cell Sorting (MACS) for EOC CSCs

CD44/CD117 antibodies conjugated to magnetic microbeads (Miltenyi Biotec, Bergisch Gladbach, Germany) were used to obtain the EOC CD44^+^CD117^+^ CSCs from HO8910 cell line. The isolation process was described in our previous reports [7, 9].

### 2.4. Serum-Free Culture Media

To obtain sphere cultures, 1 × 10^5^ murine ID8 cells of a logarithmic growth phase were seeded into 6-well plates in serum-free media supplemented with 20 *μ*g/L epidermal growth factor, 20 *μ*g/L fibroblast growth factor basic, 100 units/mL penicillin G sodium, and 100 *μ*g/mL streptomycin sulfate. After a 7-day incubation, the ID8 EOC spheres were collected, dissociated into single cell suspension by trypsin-EDTA solution and cultured to allow the regeneration of spheres. Third-generation spheres were used for subsequent experiment [17, 18].

### 2.5. Antitumor Efficacy of Vaccines in Mouse Tumor Models

The HO8910 CD44^+^CD117^+^ CSC and ID8 sphere cell lysates were achieved via repeated freezing and thawing. The experimental groups in Balb/c nude mice included the PBS, HO8910 cell, HO8910 CSC, and HO8910 non-CSC vaccination groups (six per group). The ROR1 downregulation experimental groups in Balb/c nude mice included the PBS, HO8910 cell, shROR1-HO8910 CSC, scrambled RNA- (SC-) HO8910 CSC, and HO8910 non-CSC vaccination groups (six per group). The experimental groups in C57BL/6 mice included the PBS, ID8 cancer stem-like cell (sphere cell), and ID8 noncancer stem-like cell vaccination groups (six per group). The ROR1 downregulation experimental groups in C57BL/6 mice included the PBS, shROR1-ID8 cancer stem-like cell, and SC-ID8 noncancer stem-like cell vaccination groups (six per group). The mice received subcutaneous (s.c.) vaccination in the flank in the abdomen with 2 × 10^5^ cell lysates of HO8910 CD44^+^CD117^+^ CSCs or ID8 sphere cells three times, at an interval of 10 days between adjacent immunizations. All the immunized mice were challenged s.c. with 2 × 10^6^ HO8910 or ID8 cells in 0.1 mL of PBS at 7 days after their final vaccination. Tumor growth was monitored by measuring two perpendicular tumor diameters using calipers [16, 19]. All the experiments were repeated twice.

### 2.6. Analysis of CD44^+^CD117^+^ Cell and Aldehyde Dehydrogenase- (ALDH)- Positive Cell Subsets

To detect the CD44^+^CD117^+^ cell and ALDH-positive cell subsets, the CD44 and CD117 antibodies (eBioscience, USA) and the ALDH enzyme assay kit (Invitrogen, Carlsbad, CA, USA) were applied on a flow cytometer (FCM, BD Biosciences, USA) according to the manufacturer's instructions and our previous reports [16, 19]. Briefly, a total of 2 × 10^5^ tumor cells from tumor tissues harvested from the mice immunized with different vaccines were suspended in PBS and labeled with anti-human/mouse CD44 fluorescein isothiocyanate (FITC) 1 : 100 (eBioscience, CA, USA) and anti-human CD117 phycoerythrin (PE) 1 : 20 (eBioscience, CA, USA) antibodies for immunofluorescence detection of the percentage of CSCs by a flow cytometer (FCM).

### 2.7. Cytotoxicity Assays

At the end of the animal experiments, 5 × 10^6^ splenocytes were collected from the vaccinated mice and were labeled with 0.5 mM 5-(and 6)-carboxy-fluorescein diacetate succinimidyl ester (CFSE; 20 *μ*g/mL) at 37°C for 20 min. In the NK cytotoxicity assay, the CFSE-labeled splenocytes were seeded as effector cells with a constant number of target cells (YAC-1 cells) in a 96-well plate at a 25 : 1 ratio of effector cells to target cells. In the splenocyte cytotoxicity assay, the CFSE-labeled splenocytes were placed as effector cells at a 96-well plate with a constant number of HO8910 or ID8 target cells at 25 : 1 ratios of effector cells to target cells. The cell mixtures were washed in PBS-1% BSA and incubated in a buffer containing 20 mg/mL 7-AAD (Sigma-Aldrich) for 20 min at 4°C in the dark. In the antibody-dependent cell cytotoxicity (ADCC) assay, the CFSE-labeled splenocytes and HO8910 cells were put in a 96-well plate at a 25 : 1 ratio of effector cells to target cells, and the vaccinated murine serum (1 : 100 dilution) was simultaneously put in the plate. In the complement-dependent cytotoxicity (CDC) assay, 2 × 10^5^ HO8910 and ID8 cells were plated into a 96-well plate, and the vaccinated murine serum and the guinea pig serum were added to the wells in different concentrations. The cells were incubated in a buffer containing 20 mg/mL 7-AAD (Sigma-Aldrich) for 30 min at 4°C in the dark. All the cytotoxicity assays were performed in triplicate. Flow cytometric CFSE/7-AAD cytotoxicity assay was analyzed by a FCM [20–22].

### 2.8. ELISA for Cytokines and Anti-ROR1 Antibody

The measurement of serum IFN-*γ*, TGF-*β*1, and anti-ROR-1 antibody via commercially available enzyme-linked immunosorbent assay (ELISA) was performed according to the manufacturer's protocol (eBioscience) [23, 24].

### 2.9. qRT-PCR

To evaluate the RNA expression of ROR-1, MUC-1, and NY-ESO-1, total RNAs were used for the reverse transcription (RT) reactions, and qRT-PCR was performed on a Step One Plus real-time system (AB Applied Biosystems). *β*-Actin was used as an internal control. cDNAs were amplified by PCR with the following primers: ROR-1 (sense, 5′-CATCACCACGTCTCTGGGC-3′; antisense, 5′-CTCCTTGCCGTTTGTTGCC-3′), MUC-1 (sense, 5′-CAGGTTCTTCAGG GCCAGAG-3′; antisense, 5′-TGTCCGAGAAA TT GGTGGGG-3′), NY-ESO-1 (sense, 5′-G TTCTACCTCGCCATGCCTT-3′; antisense, 5′-CTC CTTCAGAAGCACCCCTG-3′), and *β*-actin (sense, 5′-GTCCACCGCAAATGCTTCTA-3′; antisense, 5′-TGCTGTCACCTTCACCGTT C-3′) [7].

### 2.10. Construction of Recombinant Containing shRNA1 Targeting the ROR1 Gene

A pSUPER-EGFP1 (enhanced green fluorescent protein 1) vector was used to construct the recombinant pSUPER-EGFP1-ROR1-shRNA (shROR1) as previously described [7]. A pSUPER-EGFP1-scrambled shRNA (SC) was used as a negative control. These recombinants were verified by the analysis of endonuclease digestion and sequencing.

### 2.11. Western Blotting

1 × 10^6^ cells were harvested and lysed in protein extraction buffer (Novagen, Madison, WI, USA) according to the manufacturer's protocol, and the lysates were run on Western blotting as previously described [25]. The antibodies used for Western blotting included rabbit anti-human/mouse ROR1, from Abcam (Cambridge, UK), and GAPDH from BD Biosciences (Palo Alto, CA, USA), respectively.

### 2.12. Statistical Analysis

The values of interest were presented as the mean plus or minus standard deviation. Statistical comparisons were performed using the Student *t*-test method. Differences were considered statistically significant if *p* < 0.05 or lower.

## 3. Results

### 3.1. CSC Vaccination Confers Prophylactic Immunity against Tumor Cell Challenge

**Figure 1:**
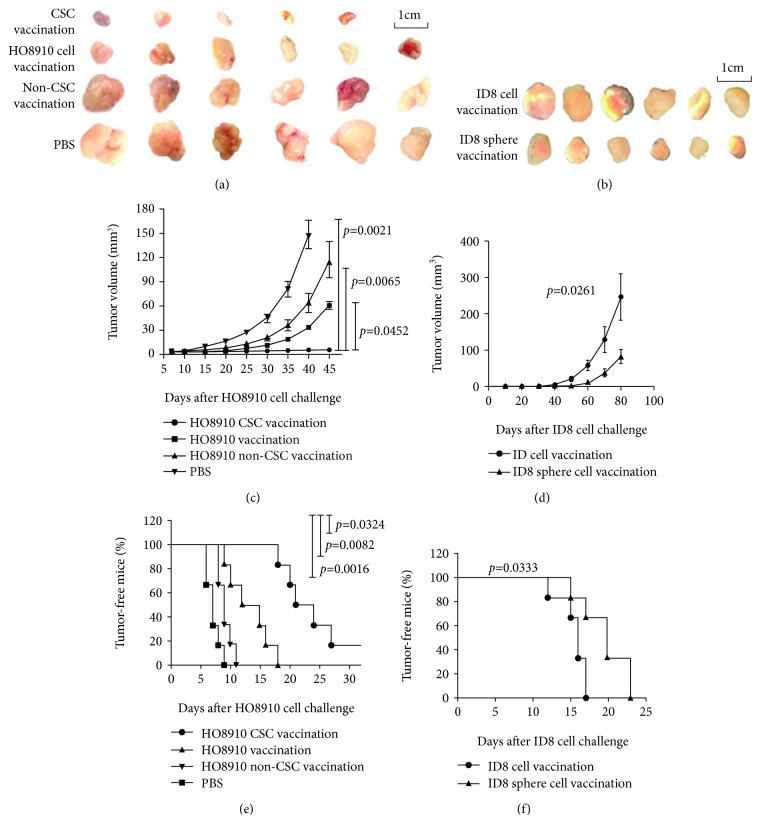
Evaluation of CSC vaccination evoking antitumor efficacy in a mouse model. (a, b) Tumor size images on day 45 from the HO8910 tumor-bearing nude mice or on day 80 from the ID8 tumor-bearing mice after mice were initially vaccinated s.c. with 2 × 10^5^ different inactivated vaccines three times with a 10-day interval, followed by a challenge with 1 × 10^6^ HO8910 or 2 × 10^6^ ID8 cells at one week after final vaccination. (c, d) Kinetic quantification of tumor sizes by measuring two perpendicular tumor diameters using calipers. (e, f) Tumor-free mice. All the data represent the mean ± SD (*n* = 6 per group; representative images). Statistically significant differences between the experiment group and the normal group were indicated.

To evaluate CSC vaccine effects, we adopted the HO8910 CD44^+^CD117^+^ CSCs and ID8 sphere cell lysates in immunizing mice three times with a 10-day interval between adjacent immunizations. At 7 days after the final immunization, mice were subcutaneously challenged with HO8910 or ID8 cells (see Materials and Methods). Figures 1(a) and 1(b) give the images of tumor sizes taken from the immunized Balb/c athymic nu/nu mice and C57BL6 mice, respectively. Remarkably, vaccination with both HO8910 CSC and ID8 cancer stem-like cell vaccines resulted in inhibiting tumor development and prolonging the tumor-bearing mouse survival. Though mice immunized with the CSC vaccines did eventually grow tumors, the sizes were much smaller than those in the mice immunized with the HO8910 cell vaccine (Figure 1(c), *p* < 0.0452) or the HO8910 non-CSC vaccine (*p* < 0.0065) or the ID8 noncancer stem-like cell vaccine (Figure 1(d), *p* < 0.0261). We found that both HO8910 CSC and ID8 cancer stem-like cell vaccinations significantly delayed tumor generation in vaccinated mice compared with any other control vaccinations as shown in Figures 1(e) and 1(f). Although the HO8910 cell-vaccinated mice did have significant protection against HO8910 cell challenge in view of the tumor forming time in the mice compared with HO8910 non-CSC-vaccinated mice, the immune prophylactic efficacy of HO8910 CSC vaccine was the best among four experimental groups (Figure 1(e)). Thus, both HO8910 CSC and ID8 cancer stem-like cell vaccinations led to the inhibition of tumor growth.

### 3.2. CSC Vaccinations Decrease CD44^+^CD117^+^ Cell and ALDH-Positive Cell Subsets

**Figure 2:**
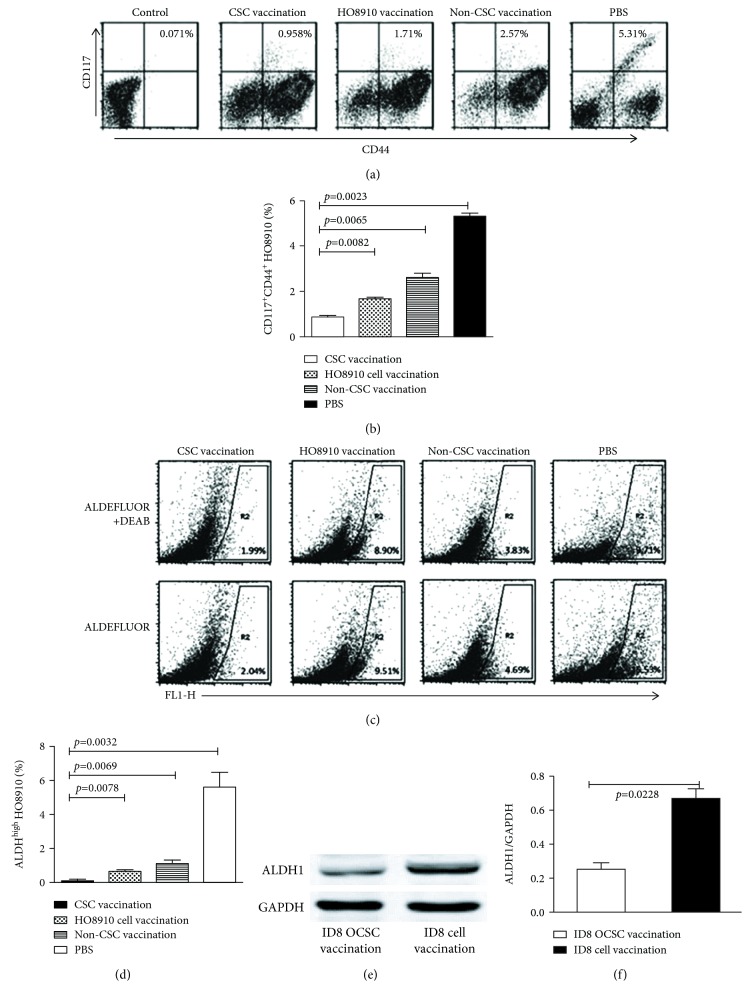
Analysis of CD44^+^CD117^+^ cell and ALDH-positive cell subsets. (a) Representative FCM plots of HO8910 CD44^+^CD117^+^ double-positive cells were accounted in various groups. (b) Quantification of CD44^+^CD117^+^ cells. (c) FCM plot of 1 × 10^6^ ovarian cancer tissue cells using the ALDEFLUOR assay. The sorting gates were established based on DEAB-stained controls. DEAB were used to establish the baseline fluorescence of these cells (R1) and to define the ALDEFLUOR-positive cell region (R2). (d) Quantitative analysis of ALDH-positive cell subset. (e) Western blot analysis of ALDH expression in ovarian cancer tissues derived from vaccinated mice. (f) Quantification of ALDH expression. Statistically significant differences were indicated.

To estimate the targeted effect of CSC vaccinations on the elimination of CSC subset, we analyzed the CD44^+^CD117^+^ cell and ALDH-positive cell subsets in ovarian cancer tissues from the CSC-vaccinated mice. Using the FCM analyses, we found that the HO8910 CD44^+^CD117^+^ double-positive cells accounted for 0.958% (Figure 2(a)) in the HO8910 CSC vaccine group, which was statistically significantly different from that of the HO8910 cell vaccine (1.71%), of the non-CD44^+^CD117^+^ CSC vaccine (2.57%), and of the PBS groups (5.31%), respectively, as shown in Figure 2(b).

ALDH1, an enzyme that is responsible for the oxidation of intracellular aldehydes, is a CSC's biomarker in a variety of cancers [26, 27]. We analyzed the ALDH1 activity in cells derived from HO8910 or ID8 EOC tissues. The data presented in Figure 2(c) shows that the ALDEFLUOR-positive cells in the CD117^+^CD44^+^ CSC vaccination group accounted for 0.05% (2.04% minus 1.99%), which was significantly reduced compared with those in the HO8910 cell vaccination group that accounted for 0.61% (9.51% minus 8.90%), non-CSC vaccination group with 0.86% (4.69% minus 3.83%), and PBS group with 5.82% (15.53% minus 9.71%), respectively. The statistical analysis results are shown in Figure 2(d). Since no commercial anti-mouse ALDH1 was available, we analyzed the ALDH1 expression in EOC tissue from vaccinated mice by Western blot assay.  Figure 2(e) shows the ALDH1 expression was significantly reduced in ID8 cancer stem-like cell-immunized mice compared to ID8 noncancer stem-like cell-immunized mice (*p* < 0.0228, Figure 2(f)). It is therefore reasonable to conclude that the CSC vaccinations could reduce the CD44^+^CD117^+^ CSC and ALDH-positive cell subsets in ovarian cancer tissues.

### 3.3. CSC Vaccinations Potentiate the Cytolytic Capacity of NK and Spleen Cells in Vaccinated Mice

**Figure 3:**
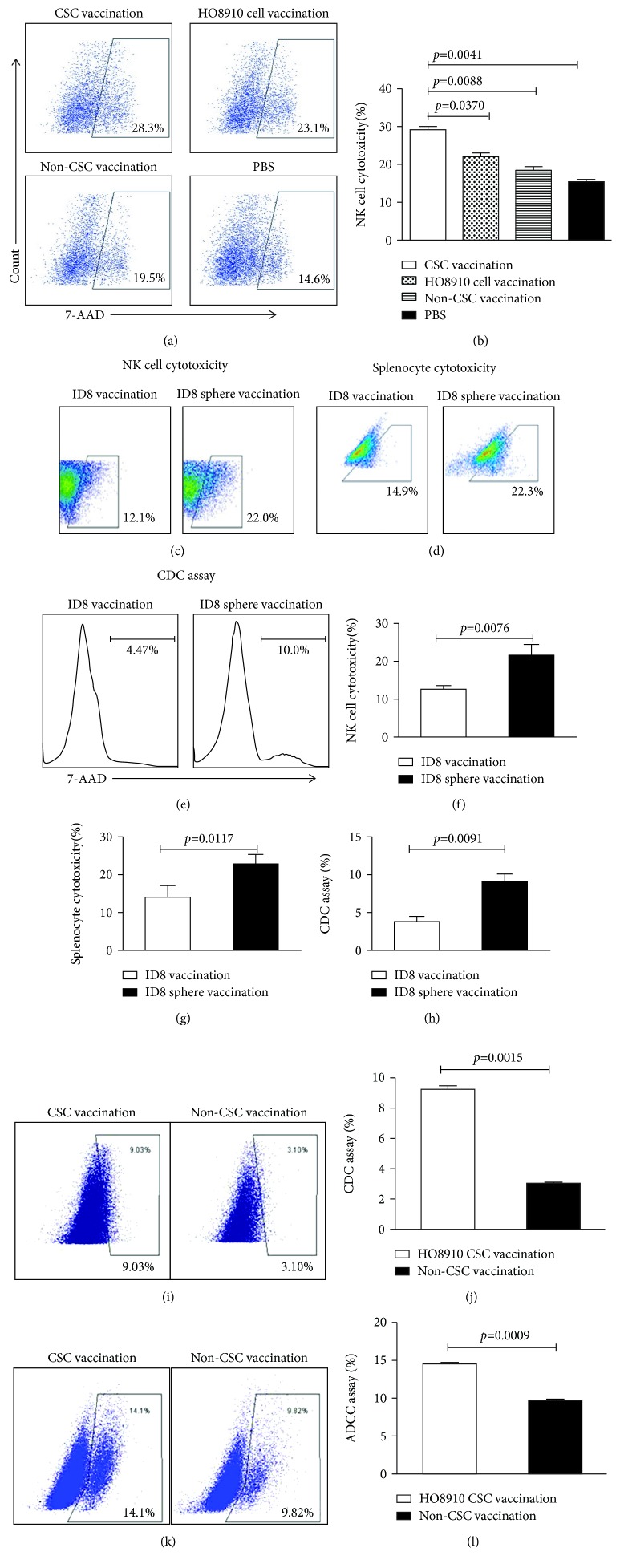
Detection of the cytolytic activities of NK, CDC, and ADCC in the vaccinated mice. The NK cytotoxic activity (target cells: YAC-1) was analyzed by FCM in the HO8910 CSC vaccine-immunized mice (a) and the ID8 sphere vaccine-immunized mice (c). (b, f) Quantification of NK cytotoxic activity. (d) The splenocyte cytotoxicity (target cells: ID8) was analyzed by FCM in the ID8 sphere vaccine-immunized mice. (g) Quantification of the splenocyte cytotoxicity. (e) FCM analysis of the CDC activity. (h) Quantification of CDC activity in ID8 vaccines. (i) FCM analysis of the CDC activity in the HO8910 CSC vaccine-immunized mice. (j) Quantification of CDC activity in HO8910 vaccines. (k) FCM analysis of the ADCC activity. (l) Quantification of ADCC activity in ID8 vaccines. All the data represent the mean ± SD (*n* = 12). Statistically significant differences between the different groups were indicated.

To evaluate whether CSC vaccination could increase the cytolytic capacity of immune cells, we first detected the NK cytotoxic activity in vaccinated mice. Figures 3(a) and 3(c) indicate that the NK cytotoxic activity was higher in both the HO8910 CD117^+^CD44^+^ CSC and ID8 cancer stem-like cell vaccination groups than that in the control vaccination groups, and the difference was statistically significant as shown in Figures 3(b) and 3(f).  Figure 3(d) indicates that the splenocyte cytotoxicity was significantly increased in the ID8 cancer stem-like cell vaccination group compared to the control group (Figure 3(g), 22.3% vs. 14.9%, *p* < 0.0117). Since immune sera from CSC-vaccinated mice contained a high level of IgG bound to CSCs, resulting in CSC lysis in the presence of complement [28, 29], we next measured the CDC activity. Notably, the CDC activity in the ID8 cancer stem-like cell- and HO8910 CD117^+^CD44^+^ CSC-vaccinated groups was significantly higher than that of the control vaccinated groups as shown in Figures 3(e) and 3(h) (10.00% vs. 4.47%, *p* < 0.0091) and Figures 3(i) and 3(j) (9.03% vs. 3.10%, *p* < 0.0015). In addition, the ADCC activity was as well significantly increased in the HO8910 CSC-vaccinated group compared with the non-HO8910 CSC-vaccinated group (Figures 3(k) and 3(l), 14.1% vs. 9.82%, *p* < 0.0009).

### 3.4. CSC Vaccinations Increase IFN-*γ* and Anti-ROR1 Antibody Levels but Decrease TGF-*β* Level in Immunized Mice

**Figure 4:**
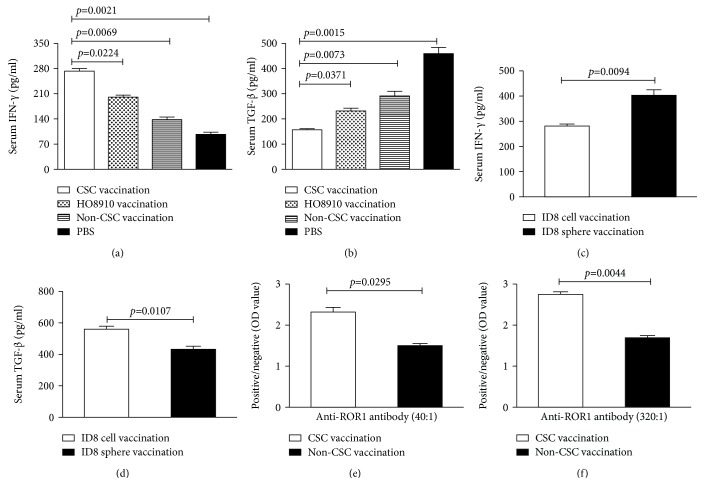
The levels of IFN-*γ*, anti-ROR1 antibody, and TGF-*β*1 as measured by ELISA. (a, c) Serum IFN-*γ* level in the different vaccinated groups. (b, d) Serum TGF-*β*1 level in the different vaccinated groups. (e, f) Anti-ROR1 antibody level in the second (serum 1 : 40 dilution) and the third (serum 1 : 320 dilution) immunizations. Data are represented as the mean ± SEM (*n* = 12). Statistically significant differences were indicated.

To further investigate the immune mechanisms underlying antitumor immunity in CSC-vaccinated mice, we measured the levels of interferon *γ* (IFN-*γ*) (Figures 4(a) and 4(c)), TGF-*β* (Figures 4(b) and 4(d)), and the anti-ROR1 (Figures 4(e) and 4(f)) using ELISA. The results showed that the IFN-*γ* level was significantly increased in the HO8910 CSC vaccination group compared with the HO8910 cell vaccination (*p* < 0.0224), the non-CSC vaccination (*p* < 0.0069), and the PBS vaccination groups (*p* < 0.0021). The similar trend was observed in mice immunized with ID8 cancer stem-like cells (Figure 4(c), *p* < 0.0094). On the other hand, TGF-*β* production was found to be notably decreased in both HO8910 CSC- and ID8 cancer stem-like cell-vaccinated mice; the differences were statistically significant (Figures 4(b) and 4(d)). Figures 4(e) and 4(f) exhibit the anti-ROR1 antibody level in both the HO8910 CSC- and non-HO8910 CSC-vaccinated groups after the second immunization (Figure 4(e)) and the third immunization (Figure 4(f)).

### 3.5. Suppressing ROR1 Expression Decreases CSC Vaccine Immunogenic and Prophylactic Efficacy

**Figure 5:**
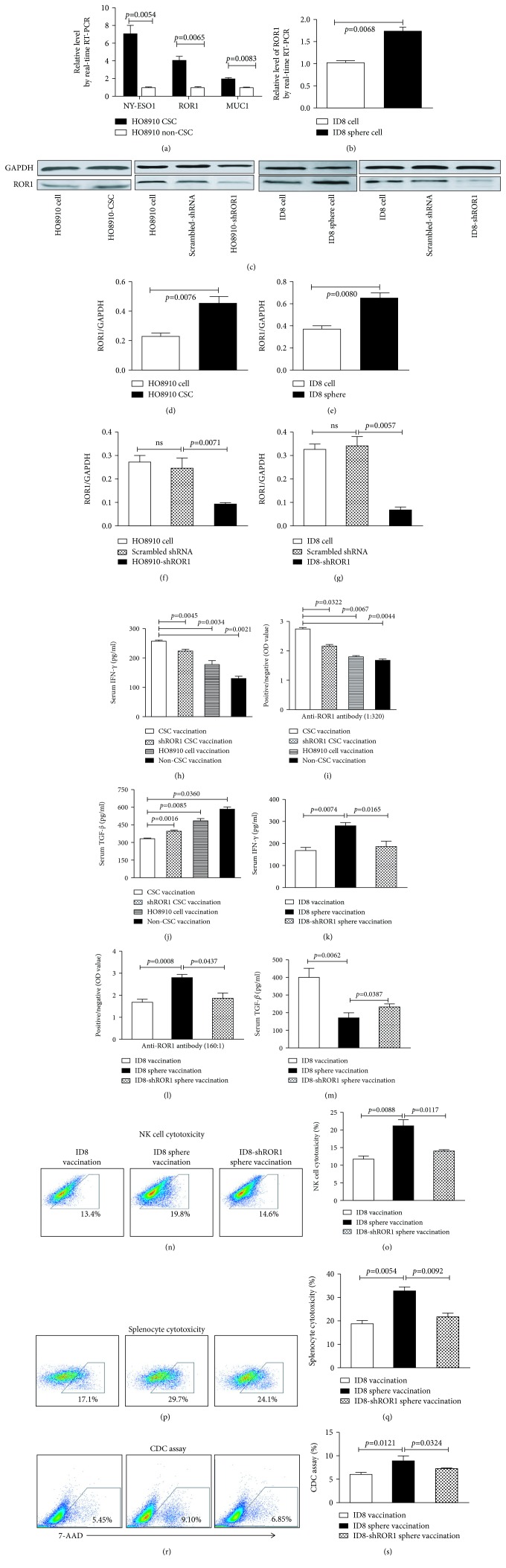
Vaccination-associated predominating antigen expressions and effects of ROR1 knockdown on immunogenicity of CSC vaccination. (a) The mRNA expressions of ROR-1, MUC-1, and NY-ESO-1 in HO8910 CSCs and HO8910 non-CSCs detected by qRT-PCR. (b) ROR-1 expression in the different cells detected by qRT-PCR. (c–g) ROR-1 expression in the different cells analyzed by Western blotting and quantification of analysis. (h) Serum IFN-*γ* level in shROR1 HO8910 CSC-vaccinated mice. (i) Anti-ROR1 antibody level in the shROR1 HO8910 CSC-vaccinated mice (serum 1 : 320 dilution). (j) Serum TGF-*β*1 level in shROR1 HO8910 CSC-vaccinated mice. (k) Serum IFN-*γ* level in shROR1 ID8 sphere-vaccinated mice. (l) Anti-ROR1 antibody level in the shROR1 ID8 sphere-vaccinated mice (serum 1 : 160 dilution). (m) Serum TGF-*β*1 level in shROR1 ID8 sphere-vaccinated mice. (n) NK cytotoxicity in shROR1-ID8 sphere-vaccinated mice. (o) Quantification of NK cytotoxicity. (p) Splenocyte cytotoxicity in shROR1-ID8 sphere-vaccinated mice. (q) Quantification of splenocyte cytotoxicity. (r) CDC activity in shROR1-ID8 sphere-vaccinated mice. (s) Quantification of CDC activity. Statistically significant differences between the different groups were indicated.

A lingering question for us in the study was why the CSC vaccinations induced stronger immune response than non-CSC vaccinations? To address this, we first selectively measured the immune-related antigen expressions in CSCs and non-CSCs by qRT-PCR analyses.  Figure 5(a) shows that the expressions of NY-ESO-1, ROR-1, and MUC-1 were significantly higher in human HO8910 CSCs than those in HO8910 non-CSCs, which were statistically significant (*p* < 0.0054, *p* < 0.0065, and *p* < 0.0083). The similar expression of ROR-1 was observed in ID8 sphere cells (Figure 5(b), *p* < 0.0068). High interest in ROR1-enriched predominating antigen epitopes made us to next detect the ROR-1 protein expression. ROR1 expression was indeed higher in HO8910 CSCs and ID8 cancer stem-like cells (sphere cells) than that in non-CSCs and ID8 cells analyzed by Western blot (Figures 5(c)–5(e)). The results suggest that the high expression of immune-related antigen ROR-1 in CSCs may be one of factors that potentiate CSC vaccination to elicit a powerful immune prophylactic efficacy.

To verify this hypothesis, we wanted to know whether suppressing ROR1 expression would decrease CSC vaccine immunogenicity and prophylactic efficacy. Figures 5(c), 5(f), and 5(g) indicate that the ROR1 expression was significantly lessened in shROR1-HO8910 CSCs and shROR1-ID8 cancer stem-like cells compared with that in the HO8910 CSCs and ID8 cancer stem-like cells. The decreased expression of ROR1 significantly reduced the levels of IFN-*γ* and anti-ROR1 antibody but enhanced the TGF-*β* level in shROR1-HO8910 CSC-vaccinated mice compared to those in the HO8910 CSC-vaccinated mice (Figures 5(h)–5(j)) and in the ID8 cancer stem-like cell-vaccinated mice (Figures 5(k)–5(m)). In addition, the activities of NK cells (Figures 5(n) and 5(o)), splenocytes (Figures 5(p) and 5(q)), and CDC (Figures 5(r) and 5(s)) were also reduced in shROR1-ID8 cancer stem-like cell-vaccinated mice.

**Figure 6:**
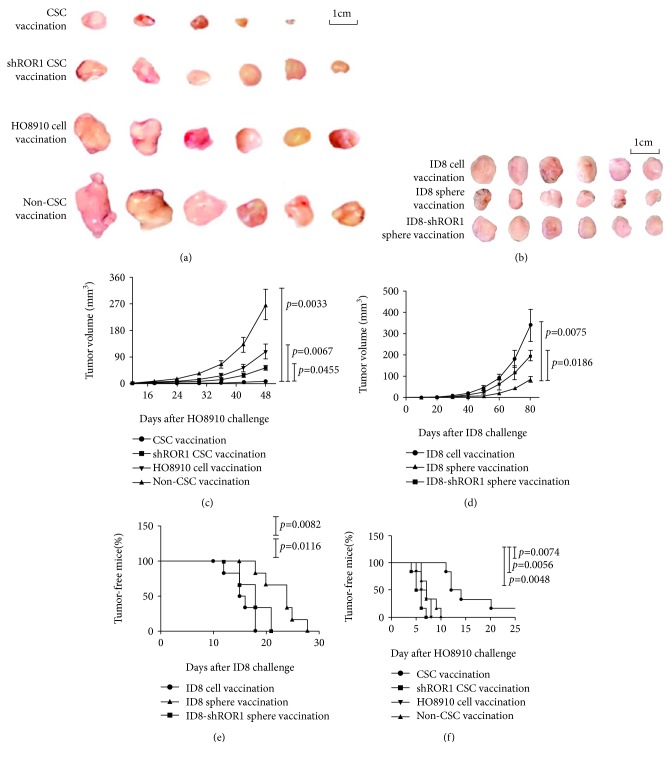
The shROR1 CSC vaccination decreased the antitumor ability. (a) Tumor size images on day 48 from the HO8910 tumor-bearing nude mice immunized initially with the 2 × 10^5^ shROR1 HO8910 CSC and other control vaccines. (b) Tumor size images on day 80 from the ID8 tumor-bearing mice immunized with the 2 × 10^5^ shROR1 ID8 cancer stem-like cell and other control vaccines. A total of three times, with an interval of 10 days between the immunizations, followed by a challenge with 1 × 10^6^ HO8910 cells (a) or 2 × 10^6^ ID8 cells (b) at one week after final vaccination. (c, d) Kinetic quantification of tumor sizes. (e, f) Tumor-free mice. All the data represent the mean ± SD (*n* = 6 per group; representative images). Statistically significant differences between the different groups were indicated.

Consistent with their reducing immunogenicity, both shROR1-HO8910 CSC and shROR1-ID8 cancer stem-like cell vaccinations significantly increased tumor sizes compared with HO8910 CSC vaccination (Figures 6(a) and 6(c)) and ID8 cancer stem-like cell vaccinations (Figures 6(b) and 6(d)), respectively. We found the similar tendency of accelerated tumor formation revealed in both shROR1 HO8910 CSC- and shROR1 ID8 cancer stem-like cell-vaccinated mice (Figures 6(e) and 6(f)). These findings demonstrated that knockdown of *ROR1* gene decreased the dominating antigen ROR1expression in CSCs, resulting in attenuating the CSC vaccinal prophylactic efficacy.

## 4. Discussion

Cancer vaccines could utilize the immune system in preventing tumor further malignant growth with immune response selectively targeted toward malignant tumor cells [29, 30]. Therefore, cancer prevention via prophylactic and therapeutic vaccines in tumor immunotherapeutic approach has gained considerable attention [31, 32]. In an effort to understand whether the immune response exerts a role in controlling ovarian cancer, we isolated the HO8910 CD44^+^CD117^+^ CSCs from the human EOC HO8910 cell line [7, 9, 16] and selected the ID8 suspension sphere cells, which were presumed as cancer stem-like cells, from murine EOC ID8 cell line [17, 18], and used them as prophylactic CSC vaccines to investigate the antitumor immunity efficacy and mechanisms.

Our data demonstrated that the CSC vaccines significantly increased the anti-ROR1 antibody and IFN-*γ* levels but reduced the TGF-*β* level as well as increased the cytotoxicities of NK cells, splenocytes, and CDC compared to the non-CSC vaccines. Both HO8910 CD44^+^CD117^+^ cell and ID8 cancer stem-like cell vaccinations provided significant protection against the tumor cell challenges and inhibited the growth of tumor cells as xenografts in nude mice or as allografts in C57BL/6 mice compared to the control vaccinations. The efficiency was reflected in delaying the tumor formation time and extending tumor-bearing mouse survival in both Balb/c nude mice and C57BL/6 mice.

In the present study, we used the repeated freeze-thaw whole HO8910 CD44^+^CD117^+^ cell and ID8 suspension sphere cells instead of using dendritic cells (DCs) loaded with CSC lysates to prepare CSC vaccines [33, 34] and then directly injected the CSC lysates to mice. This is because the developed CSC vaccines by a repeated freeze-thaw way could induce CSC necrosis and release numerous danger signals such as DNA, RNA, uric acid, and heat-shock proteins that evoke partial DC maturation in vivo [34]. Uric acid and heat shock proteins could bind to scavenger receptor-A and toll-like receptor 4 on DCs and allow DCs to present tumor antigens to elicit immune responses [35, 36]. Consequently, our developed vaccines induced a powerful antitumor immunity against EOC growth.

According to the results of animal experiments, we wanted to know why CSC immunization effectively confers protection against EOC development whereas the same non-CSC immunization is only partially effective in the reduction of tumor burden. As far as we know, CSCs contribute to tumor growth, metastasis, and relapse due to their relative resistance to chemotherapy and radiation therapy following treatment [18, 37, 38]. We hypothesized that the immune responses induced by CSC-based vaccines might preferentially target the CSC subpopulation for inhibiting tumor growth. To verify it, we detected the HO8910 CD44^+^CD117^+^ CSC subpopulation in xenograft tumor tissues. As we hypothesized, the number of HO8910 CD44^+^CD117^+^ CSCs was markedly reduced in EOC tissues from the CSC-vaccinated mice in comparison with the non-CSC-vaccinated mice. The result was further supported by findings with ALDH1 assay. This is because the ALDH1-positive cells represent CSC features and are usually used for a biological biomarker of CSC [16, 26, 27]. Like the CD44^+^CD117^+^ CSC counts, the ALDH1 cell counts was concurrently decreased in EOC tissues from the CSC-vaccinated mice. Based on these data, we concluded that CSC vaccination-induced anti-EOC immunity could directly target CSCs to reduce the CSC subpopulation.

To understand the molecular mechanisms involved CSC vaccine antigens, we selectively screened tumor-associated predominant antigens in CSC vaccines. ROR-1, as detected immunologically, is a conserved carcinoembryonic surface antigen. ROR-1 expression is found to increase in numerous blood and solid malignancies, but is very low in healthy adult tissues; these properties of ROR-1 make it a potential target for cancer immunotherapy [8, 39, 40]. We found ROR-1 was indeed highly expressed in human HO8910 CSCs and in ID8 cancer stem-like cells. If ROR-1 is one of the dominating antigens in CSC vaccine, the diminution of ROR1 expression should impact the vaccine immunogenic and prophylactic efficacy. Indeed, the prophylactic effects of CSC vaccines in both HO8910 CSC- and ID8 cancer stem-like cell-vaccinated mice were attenuated by ROR1 downregulation in CSCs. This revealed that the levels of IFN-*γ* and anti-ROR1 were decreased whereas TGF-*β* level was enhanced in shROR1-HO8910 CSC-vaccinated mice. It is known that IFN-*γ* activates the splenocyte and NK cells and not only improves their cytotoxic activity but also increases IFN-*γ* secretion by NK cells that play important roles in killing tumor cells by ADCC mechanism in the antitumor immunity [7, 17, 18], whereas the malignant tumors secrete high amounts of TGF-*β* that is associated with the advanced stage of the tumors, thereby promoting tumor growth and lessening patient survival [7, 15, 19]. Accordingly, these findings may support our hypothesis that CSCs enriched by virtue of their expression of the ROR1 were more immunogenic and effective than non-CSCs.

To further evaluate the antitumor immunity mechanisms mediated by CSC-based vaccines, we employed murine EOC ID8 cell line to isolate CD117^+^CD44^+^ CSCs for the investigation. Unfortunately, the subpopulations are very rare or even undetectable when we tried to identify them by using a magnetic-activated cell sorting system or fluorescence-activated cell sorting. We alternatively selected the suspension tumor sphere cells, i.e., CSC-enriched population as murine EOC CSCs for this investigation. This was because the suspension tumor sphere cells acquired from serum-free culture were supposed to be highly related to cancer stem-like cell characteristics such as chemoresistance, radioresistance, high soft agar clone formation capability, and tumorigenicity; these are essentially consistent with the previous investigations by others [41–43] and by us [17, 44, 45]. In contrast to Balb/c athymic nu/nu mice, C57BL/6 mice were of an appropriate animal model for objectively appraising the immunogenicity of the ID8 cancer stem-like cell vaccine. With the growth of the ID8 cell-transplantable EOC being suppressed, the cytotoxic activities of splenocytes, NK cells, and CDC as well as serum anti ROR-1 antibody and IFN-*γ* levels were increased in the ID8 cancer stem-like cell-vaccinated mice, suggesting elicit effective immunity against EOC. Our results also revealed that attenuated splenocyte, NK, and CDC activities along with a little bit low IFN-*γ* and high TGF-*β*1 levels were found in shROR1-ID8 cancer stem-like cell-vaccinated mice. This further suggested that the ROR-1 was apparently associated with an immunogenicity in CSC vaccinations.

In the present study, we also evaluated if whole CSC lysates would trigger immune responses to various self-antigens. In this regard, we used the histopathology and blood routine test to analyze liver and kidney function and blood cell changes. The results indicated that there was no presence of any lung and liver tissue injuries as well as blood cell and kidney functional abnormality mediated by immune response (data not shown here), suggesting our development of CSC vaccines is safe.

The adaptable nature of CSC vaccination is partially governed by the CSC antigen nature. Although we have identified the ROR-1 enriched by predominating antigen epitopes in CSCs, a better knowledge of other predominating and neonatal antigens in CSCs remains a challenge for guiding CSC immunotherapy in our further investigation.

## 5. Conclusions

In conclusion, our study findings strongly suggest that elimination of tumor cells evoked by CSC vaccination used in the present study was mediated by selectively recognizing and eradicating CSCs. CSCs were enriched by virtue of high expression of the dominant antigen ROR-1 contribute to its immunogenicity and confer effectively antitumor immunity. The findings should encourage the development of CSC-based vaccination aiming to eliminate or reduce the CSC subpopulation and to reinforce immunotherapeutic effects on EOC.

## Data Availability

The data used to support the findings of this study are available from the corresponding author upon request.
